# Subchronic Oral Bromocriptine Methanesulfonate Enhances Open Field Novelty-Induced Behavior and Spatial Memory in Male Swiss Albino Mice

**DOI:** 10.1155/2013/948241

**Published:** 2012-12-06

**Authors:** Olakunle James Onaolapo, Adejoke Yetunde Onaolapo

**Affiliations:** ^1^Department of Pharmacology, Ladoke Akintola University of Technology, PMB 5000, Ogbomoso, Nigeria; ^2^Department of Human Anatomy, Ladoke Akintola University of Technology, PMB 5000, Ogbomoso, Nigeria

## Abstract

This study set out to assess the neurobehavioral effects of subchronic, oral bromocriptine methanesulfonate using the open field and the Y-maze in healthy male mice. Sixty adult Swiss albino mice were assigned into three groups. Controls received normal saline, while test groups received bromocriptine methanesulfonate at 2.5 and 5 mg/kg/day, respectively, for a period of 21 days. Neurobehavioral tests were carried out on days 1 and 21 after administration. Open field assessment on day 1 after administration revealed significant increase in grooming at 2.5 and 5 mg/kg, while horizontal and vertical locomotion showed no significant changes. Day 1 also showed no significant changes in Y-maze alternation. On day 21, horizontal locomotion, rearing, and grooming were increased significantly at 2.5 and 5 mg/kg doses after administration; also, spatial memory was significantly enhanced at 2.5 mg/kg. In conclusion, the study demonstrates the ability of oral bromocriptine to affect neurobehavior in normal mice. It also suggests that there is a cumulative effect of oral bromocriptine on the behaviors studied with more changes being seen after subchronic administration rather than after a single oral dose.

## 1. Introduction

 Bromocriptine is an ergot derivative of ergoline and also an amide derivative of the d isomer of lysergic acid; it is a white, crystalline almost odorless powder [1]. Bromocriptine is absorbed largely from the gastrointestinal tract, having a half life of about 3.3 hours and reaching peak plasma levels within 1-2 hours after oral administration. Excretion is usually through bile and faeces [2]. Bromocriptine is a dopamine agonist that exerts its actions and properties at striatal D1 and D2 adenyl cyclase-linked dopamine receptors [3]. Bromocriptine inhibits prolactin secretion [4] and also inhibits glutamate release by reversing the glutamate GLT1 transporter [5]. Bromocriptine is used in the treatment of Parkinson's disease and has also been found valuable in the treatment of a number of endocrinologic and gynaecologic disorders [2, 6]. It also induces behavioral and hormonal changes that could last several hours following a single systemic dose [7], such behavioral changes include motor hyperactivity in animals. In May 2009, bromocriptine mesylate quick release was approved for the treatment of type 2 diabetes; it is believed to exert its antidiuretic actions from its influence on hypothalamic circadian neuronal activities thus resetting abnormally elevated hypothalamic drive for an increase in plasma glucose, free fatty acids, and triglycerides in patients with type 2 diabetes [8]. Bromocriptine also has antioxidant properties; a study evaluating its neuroprotective effects in 1-methyl-4-phenyl-1,2,3,6-tetrahydropyridine (MPTP) induces that toxicity concluded that bromocriptine blocked MPTP-induced behavioral dysfunction and also reversed glutathione and dopamine depletion [9, 10]. Bromocriptine was mostly known for its use in the management of CNS related disorders, with its increasing importance in the management of nonneurological conditions, its effects on neurobehaviour in the absence of brain pathology became important. A lot has been done studying the neurobehavioral effects of bromocriptine in rats or mice usually following single intraperitoneal injections [7, 11–13]. Little data, if any on the behavioral effects of subchronic or chronic oral bromocriptine in mice, are available. This study intends to make such data available. 

## 2. Materials and Method

### 2.1. Equipments and Apparatus

Electronic precision balance, plastic animal cages, sterile disposable syringes (1, 5, and 10 mL) and needles, cotton wool, stop watch and open field box, and Y-maze were used.

### 2.2. Reagents and Drugs

Normal saline, 5 mg bromocriptine tablets (Bromergon) (Lek Pharmaceutical and Chemicals), was grounded into fine powder, weighed and dissolved in measured volume of isotonic saline solution to get the desired concentrations. Bromocriptine at 2.5 and 5 mg/kg was administered orally using a cannula. 

### 2.3. Animals

Healthy adult Swiss albino mice purchased from the Empire Animal farms, Osogbo, Osun State, Nigeria, with weights ranging from 20 to 25 g were used. The animals were housed in plastic cages measuring 16′′ × 12′′ × 10′′ (10 mice in each cage). All animals had free access to food and water *ad libitum*. They were maintained under standard laboratory conditions, that is, a well-aerated room with alternating light and dark cycles of 12 h each and at room temperature of 25°C. The experimental protocol was approved by the Ladoke Akintola University Animal Ethics Committee. All rules applying to animal safety and care were observed.

### 2.4. Experimental Method

 A total of sixty animals were used for both studies, thirty animals for each of the experiments. The animals were randomly assigned into three groups A, B, and C. Group A was the control and received normal saline. Groups B and C received bromocriptine orally at 2.5 and 5 mg/kg daily, respectively, for a period of 21 days; the animals were exposed to the open field and the Y-maze thirty minutes after the first and last doses of either drug or vehicle.

### 2.5. Behavioral Tests

The behavioral tests were conducted in a large quite room between the hours of 8 am and 3 pm Novelty-induced behaviors were evaluated using the open field box and spatial learning and memory using the Y-maze. Behaviors were scored by the authors using a stop watch; all animals in a group were tested on the same day (10 animals per day). All events were observed manually.

#### 2.5.1. The Open Field Box

 The open field box is a rectangular area composed of a hard floor measuring 36 × 36 × 26 cm and made of white painted wood. The floor was divided by permanent red markings into 16 equal squares at the bottom. Generally, spontaneous motor activity was monitored for 30 min in the open field as described by Ajayi and Ukponmwan [14]. After treatment as explained earlier, each mouse was introduced into the field and the total locomotion (number of floor units entered with all paws), rearing frequency (number of times the animal stood on its hind limbs or with its fore limbs against the walls of the observation box or free in the air), and frequency of grooming (number of body cleaning with paws, picking of the body, and pubis with mouth and face-washing actions) within each 10 min interval were recorded. The arena was cleaned with 5% alcohol to eliminate olfactory bias and the arena was allowed to dry before introducing a fresh animal.

#### 2.5.2. Y-Maze

It is well known that spontaneous alternation is a measure of spatial working memory. The Y-maze can be used as a measure of short-term memory, general locomotor activity, and stereotypic behavior. Therefore, spontaneous alternation was assessed using a Y-maze composed of three equally spaced arms (120°, 41 cm long, and 15 cm high). The floor of each arm is made of Pyrex and is 5 cm wide. Each mouse was placed in one of the arm compartments and was allowed to move freely until its tail completely enters another arm. The sequence of arm entries is manually recorded, the arms being labeled A, B, or C. An alternation is defined as entry into all three arms consecutively, for instance, if the animal makes the following arm entries: ACB, CA, B, C, A, CAB, C, A, in this example, then the animal made 13 arm entries, 8 of which are correct alternations. The number of maximum spontaneous alternations is then the total number of arms entered minus two, and the percentage alternation is calculated as {(actual  alternations/maximum  alternations) × 100}. For each animal, the Y-maze testing was carried out for 5 minutes. The apparatus was cleaned with 5% alcohol and was allowed to dry between sessions. 

### 2.6. Statistical Analysis

All data were analyzed using one way analysis of variance (ANOVA) followed by a post hoc test (Student-Newman-Keuls) test carried out to determine the source of a significant effect. Results were expressed as mean ± S.E.M., and *P* < 0.05 is taken as accepted level of significant difference from control. 

## 3. Results

### 3.1. Effect of Bromocriptine on Horizontal Locomotion

**Figure 1:**
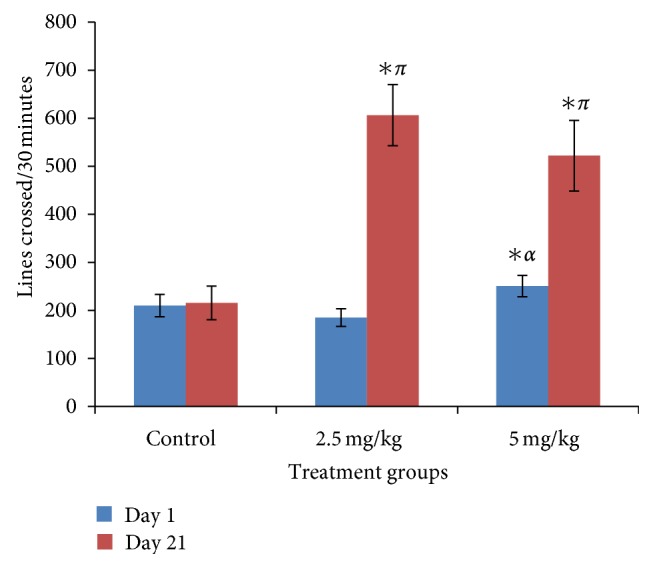
Effect of bromocriptine (2.5 and 5 mg/kg) on horizontal locomotion following 30 minutes of exploration in the open field. Each bar represents mean ± S.E.M, ^∗ *απ*^ *P* ≤ 0.05 compared to the control, *n* = 10.

Figure 1 shows the acute and subchronic effects of bromocriptine on horizontal locomotion following thirty minutes of exposure in the open field. There was a significant increase in locomotor activity at 5 mg/kg on day 1 and at 2.5 and 5 mg/kg of bromocriptine compared to control on day 21 (*f* = 11.94, *P* < 0.05, degree of freedom = 27). 

### 3.2. Effect of Bromocriptine on Rearing

**Figure 2:**
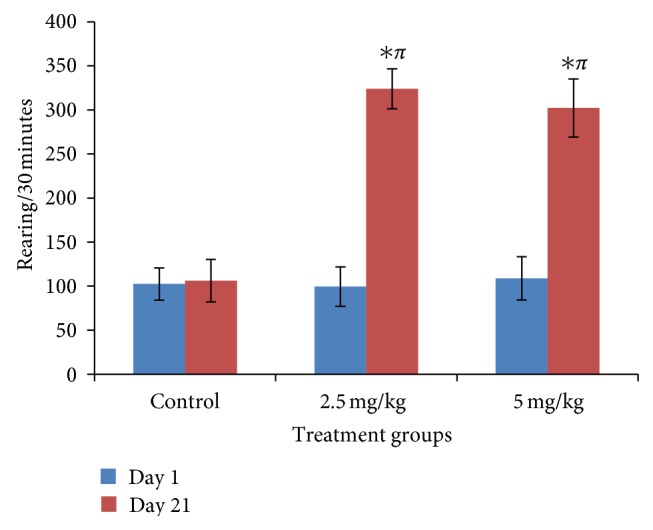
Effect of bromocriptine (2.5 and 5 mg/kg) on rearing activity following 30 minutes of exploration in the open field. Each bar represents mean ± S.E.M, ∗*P* ≤ 0.05 compared to the control, *n* = 10.

Figure 2 shows the acute and subchronic effects of bromocriptine on rearing activity following thirty minutes of exposure in the open field. On day 21, there was a significant increase in rearing activity at 2.5 and 5 mg/kg compared to control (*f* = 19.77, *P* < 0.05, degree of freedom = 27). The response seen at 2.5 mg/kg was slightly higher than that seen at the 5 mg/kg dose, although the difference was only visual.

### 3.3. Effect of Bromocriptine on Grooming Behavior

**Figure 3:**
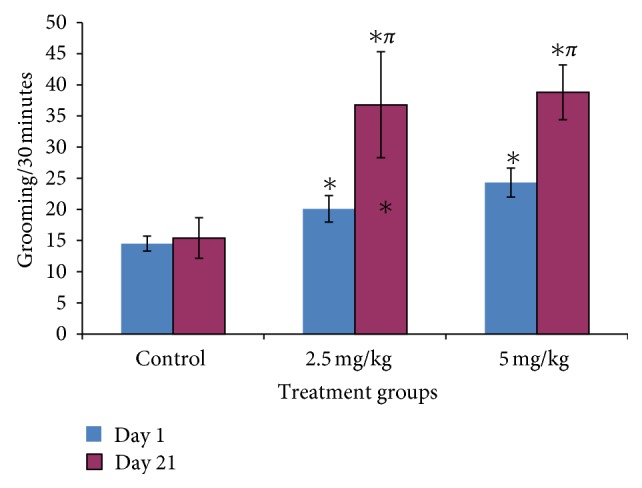
Effect of bromocriptine (2.5 and 5 mg/kg) on grooming behavior following 30 minutes of exploration in the open field. Each bar represents mean ± S.E.M, ∗*P* ≤ 0.05 compared to the control, *n* = 10.

Figure 3 shows the effects of bromocriptine on grooming behavior following thirty minutes of exposure in the open field; on both days 1 and 21, there was significant increase in grooming behaviour at 2.5 and 5.0 mg/kg of bromocriptine compared to control (*f* = 4.94, *P* = 0.015, degree of freedom = 27). 

### 3.4. Effect of Bromocriptine on Y-Maze Locomotor Activity

**Figure 4:**
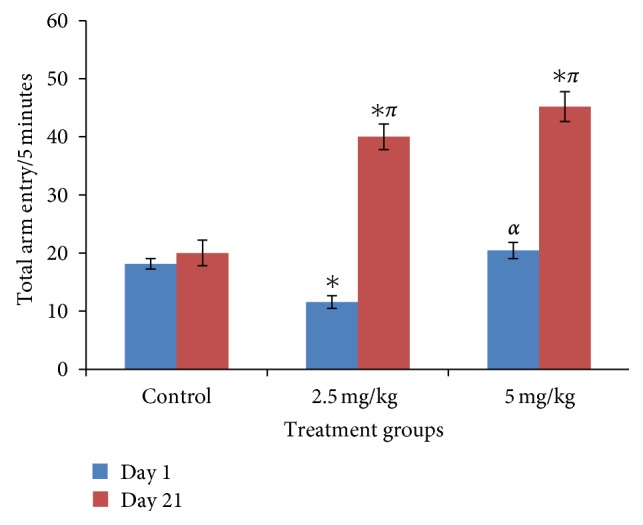
Effect of bromocriptine (2.5 and 5 mg/kg) on locomotor activity following 5 minutes of exploration in the Y maze. Each bar represents mean ± S.E.M, ^∗*απ*^ *P* ≤ 0.05 compared to the control, *n* = 10.

Figure 4 shows effects of bromocriptine on locomotor activity following 5 mins exploration in the Y-maze. On day 1, locomotor activity increased significantly at 5 mg/kg, and on day 21 it showed significant increment in locomotor activity at 2.5 and 5.0 mg/kg of bromocriptine compared to control (*f* = 32.68, *P* = 0.00, degree of freedom = 27). 

### 3.5. Effect of Bromocriptine on Spatial Memory

**Figure 5:**
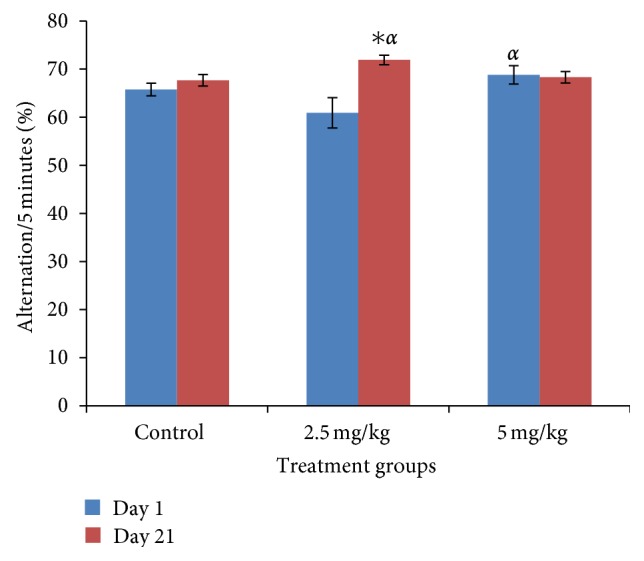
Effect of bromocriptine (2.5 and 5 mg/kg) on spatial memory following 5 minutes of exploration in the Y-maze. Each bar represents mean ± S.E.M, ∗*P* ≤ 0.05 compared to the control, *n* = 10.

Figure 5 shows the effect of bromocriptine on spatial memory following 5 mins of exploration in the Y-maze. There was a significant (*f* = 4.15, *P* = 0.027, degree of freedom = 27) increase in percentage correct alternations (spatial memory) following administration of bromocriptine at 2.5 mg/kg of bromocriptine compared to control on day 21, and comparing both doses of bromocriptine showed a more significantly increased alternation at 2.5 mg/kg than that seen at the 5 mg/kg dose.

## 4. Discussion

 Neurobehavioral effects of subchronic, oral bromocriptine in the open field, and Y-maze in male Swiss mice were studied. Initial neurobehavioral assessments after the first dose showed no significant changes in locomotion and spatial memory with only grooming behavior showing significant increases. However, results at day 21 showed that at 2.5 and 5.0 mg/kg doses compared to control, bromocriptine significantly increased both horizontal and vertical locomotor activity, and this corroborates with findings from other studies that concluded that intraperitoneal injection of bromocriptine (5–20 mg/kg) produced dose-dependent and long lasting locomotor stimulation in mice [15]. Another study reached almost the same conclusion, reporting that subcutaneous injection of bromocriptine at 3.0 mg/kg caused dose-specific elevation of locomotion in 1-methyl-4-phenyl-1,2,3,6-tetrahydropyridine- (MPTP-) treated neonatal mice; suppression of activity was, however, seen at higher doses [9, 10]. Worthy of note is the fact that although almost similar conclusions were reached by these studies, different routes of administration were used, showing that either as an injection or daily oral bolus, bromocriptine increased locomotor activity. Bromocriptine is known to produce a biphasic behavioral effect in mice, an early depression followed by stimulation [16, 17]. In this study, day 1 tests showed no significant changes except for grooming, while day 21 tests showed major changes, and changes seen on day 21 are probably attributable to accumulation of bromocriptine following repeated doses, although we intend to evaluate this in subsequent studies. Bromocriptine enhances locomotor activity by a complex involvement of both noradrenaline and dopamine pre- and postsynaptic neurons, possibly due to its partial agonist action or as a result of its active metabolite, these conclusions were deduced from an observation that the increased locomotor activity induced by bromocriptine was suppressed by drugs inhibiting both dopaminergic and noradrenergic pre- and post-synaptic actions [17].

Pelage cleaning in laboratory rodents can be seen following exposure to novelty [18]. In the study, both acute and subchronic bromocriptine significantly increased novelty-induced grooming activity at 2.5 and 5 mg/kg. A higher intensity of grooming is, however, observed with chronic administration, further suggesting that bromocriptine effects on grooming tend to increase as cumulative doses are given. It is known that central dopaminergic activation induces intense grooming via D1 receptors [19] and bromocriptine is a dopamine agonist at D1 receptors. Since we now know from this study that both acute and subchronic doses affect grooming, a part of what we intend to continue to study is the differential modulatory effect of acute versus subchronic oral bromocriptine on D1 receptors.

The Y-maze is a behavioral model that can be used to investigate locomotor activity as well as learning and memory. In this study, it was observed that Y-maze locomotor activities was significantly increased at 2.5 and 5.0 mg/kg of subchronic bromocriptine, and this is in accordance with bromocriptine ability to enhance locomotor activity as earlier observed. In the Y-maze spatial memory task, a small nonsignificant decline was seen with acute dosing at 2.5 mg/kg but at 5 mg/kg; it returned back to baseline, however, subchronic bromocriptine caused a dose-dependent change in spatial memory with peak (significant) value seen at 2.5 mg/kg and a decline at 5 mg/kg. The results we got from acute administration is consistent with previous studies reporting that bromocriptine causes dose-dependent differential effects on learning; rats that received bromocriptine at 5 mg/kg intraperitoneal injection demonstrated impairment in learning, while bromocriptine at 10 mg/kg and vehicle-treated groups did not differ from normal controls [20]. In subchronic doses, however, the ability of oral bromocriptine to enhance spatial memory at one of the doses administered is demonstrated. Studies have shown that bromocriptine is a modulator of learning and memory, as it has been shown to cause a reduction in working memory errors in mice following exposure to the 12 arm radial maze [21], and this is also true in humans [22, 23]. Bromocriptine modulates learning and memory via its mixed agonist antagonist actions at dopamine receptors with the abilities to both increase and deplete dopamine levels as the case may be [20]. 

## 5. Conclusions

 This study demonstrates the ability of oral bromocriptine to affect neurobehavior in normal mice. It also suggests that there is a cumulative effect of oral bromocriptine on the behaviors studied with more changes being seen after subchronic administration rather than after a single oral dose.
